# Supplementary material for: Liquid condensation of reprogramming factor KLF4 with DNA provides a mechanism for chromatin organization
Source: Nat Commun. 2021 Sep 22;12:5579. doi: 10.1038/s41467-021-25761-7 (PMC8458463; doi:10.1038/s41467-021-25761-7)
Supplement: Supplementary file 1 — Supplementary Information [file 41467_2021_25761_MOESM1_ESM.pdf]

Liquid condensation of reprogramming factor KLF4 with DNA provides a mechanism for chromatin organization

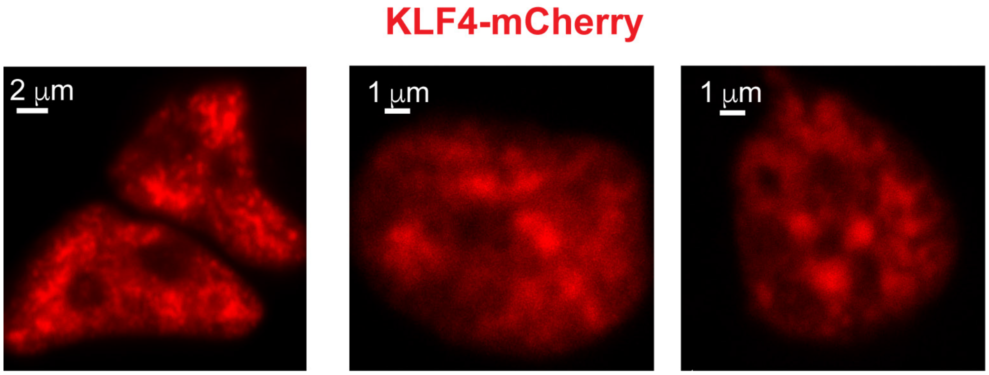

Supplementary Figure 1. **KLF4-mCherry forms a condensed liquid phase in HEK 293T cells.** Fluorescence microscopy shows that KLF4-mCherry expressed in HEK 293T cells exhibits a punctate distribution, as well as some small and large droplets. Because this morphology is similar to that of KLF4-mTurq in Figure 1, the observed biomolecular condensation is independent of the fusion tag.

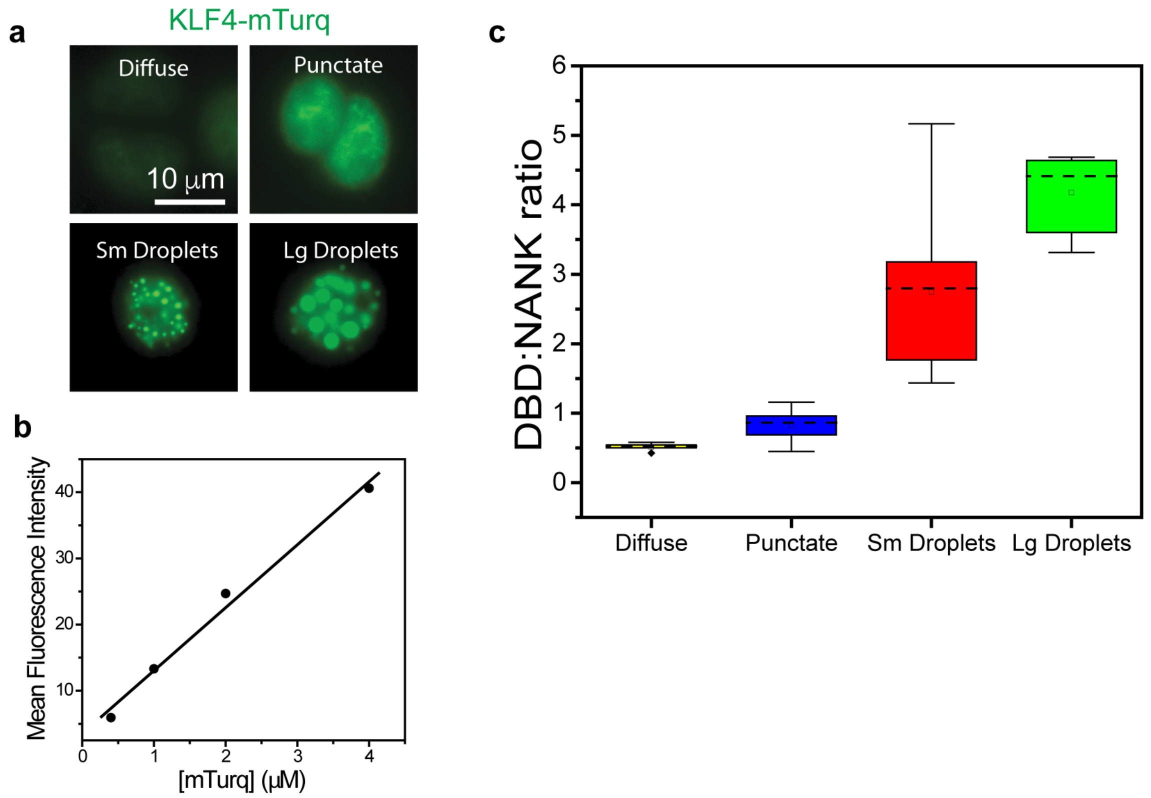

Supplementary Figure 2. **KLF4-mTurq concentrations in HEK 293T cell nuclei correlate with distributions indicative of biomolecular condensation. a** Fluorescence microscopy of KLF4-mTurq expressed in HEK 293T cells reveals nuclear distributions that we classify as diffuse, punctate, small droplets (circularity >0.8 and diameter 0.3-1 μm), or large droplets (diameter >1 μm). The four images are at the same scale. **b** Calibration of KLF4-mTurq nuclear concentrations referenced against known concentrations of purified mTurquoise2 fluorescent protein. The mean fluorescence intensities of the nuclei (in arbitrary units) were quantified using FIJI ImageJ. Plots of power/exposure time versus concentrations of purified mTurquoise2 (mTurq) fluorescent protein were linearly fit using Origin. Using an EVOS FL imaging system (Thermo Fisher Scientific), the power and exposure time settings applied were CFP filter at 30% power and 15-ms exposure time (KLF4-mTurq). **c** Quantifying KLF4-mTurq concentrations shows that cells classified as 'diffuse' express KLF4-mTurq at 0.51 ± 0.06 μM (n=5); those classified as 'punctate' express KLF4-mTurq at 0.74 ± 0.2 μM (n=11); those with small LLPS droplets (cells with at least one or two circular droplets of circularity >0.8 and diameter 0.3-1 μm) express KLF4-mTurq at 2.47 ± 1.1 μM, and those with large LLPS droplets (diameter >1 μm) express KLF4-mTurq at 4.29 ± 0.59 μM (n=6). The number of cells in the field that were classified with the indicated phenotype is given by 'n'. Whiskers indicate the full range of the data, and boxes indicate the quartiles (25% to 75%). Dashed lines indicate the median value, open squares indicate the mean value, and the black diamond is an outlier, defined as more than 1.9 interquartile distances from the median.

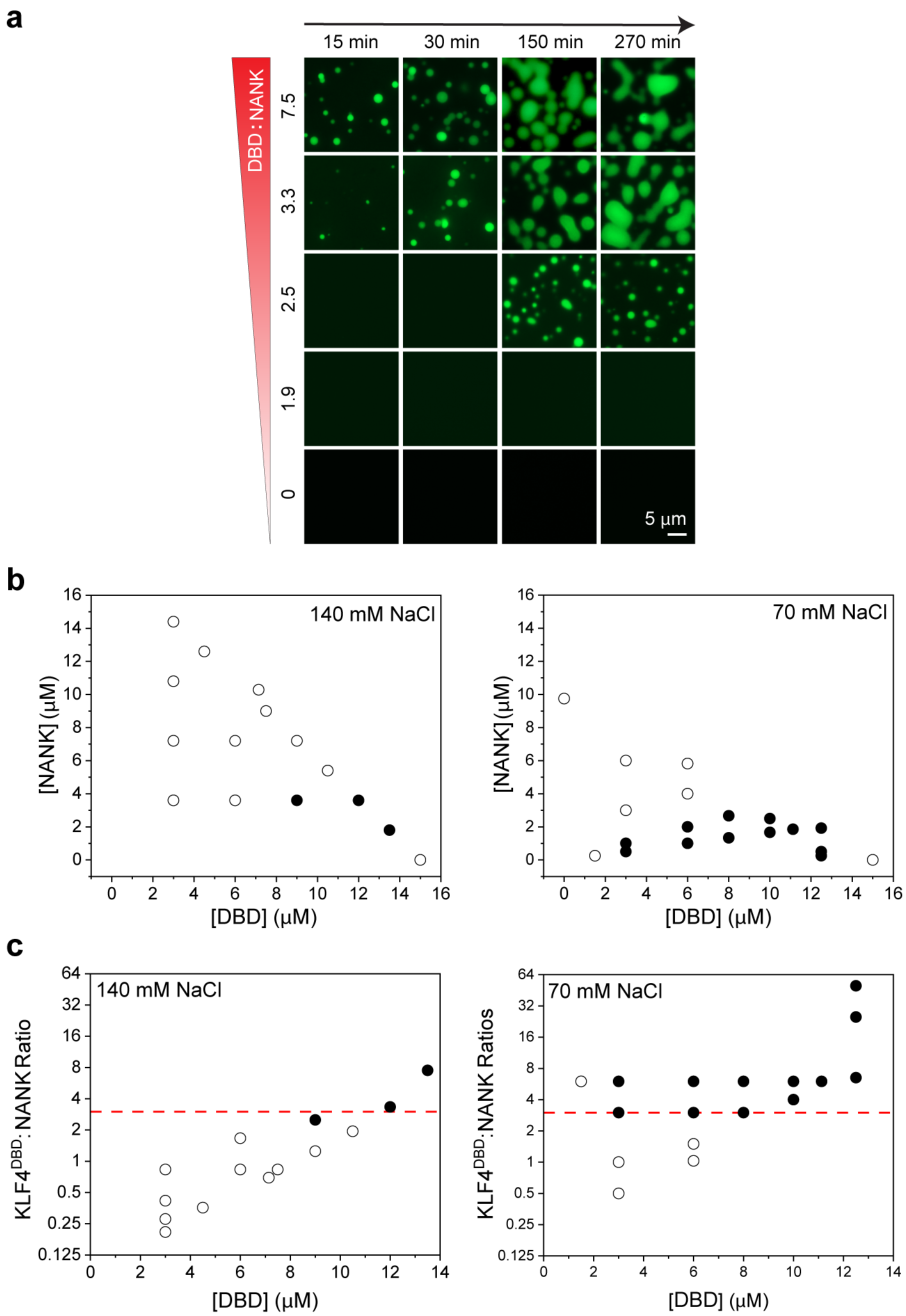

Supplementary Figure 3. **DBD liquid-liquid phase separation with the NANK duplex requires a DBD:NANK ratio close to 3:1. a** Time course of DBD:NANK droplet formation and wetting of the surface (TS2 buffer conditions with 100 nM YOYO-1). **b** LLPS diagram of DBD:NANK at various concentrations and 140 mM NaCl (TS2 buffer; left) or 70 mM NaCl (TS buffer; right). Solid black circles indicate LLPS (droplets detected at 30 min timepoint). **c** Data from **b** replotted with the ordinate axis expressed as the DBD:NANK ratio. LLPS was observed at DBD:NANK ratios of 3 and above (dashed red line) in both 140 mM NaCl (left) and 70 mM NaCl (right) solution conditions. Three DBD proteins can bind specifically to the three KLF4 cognate sites in one NANK duplex (see Figure 3A). All images are at the same scale.

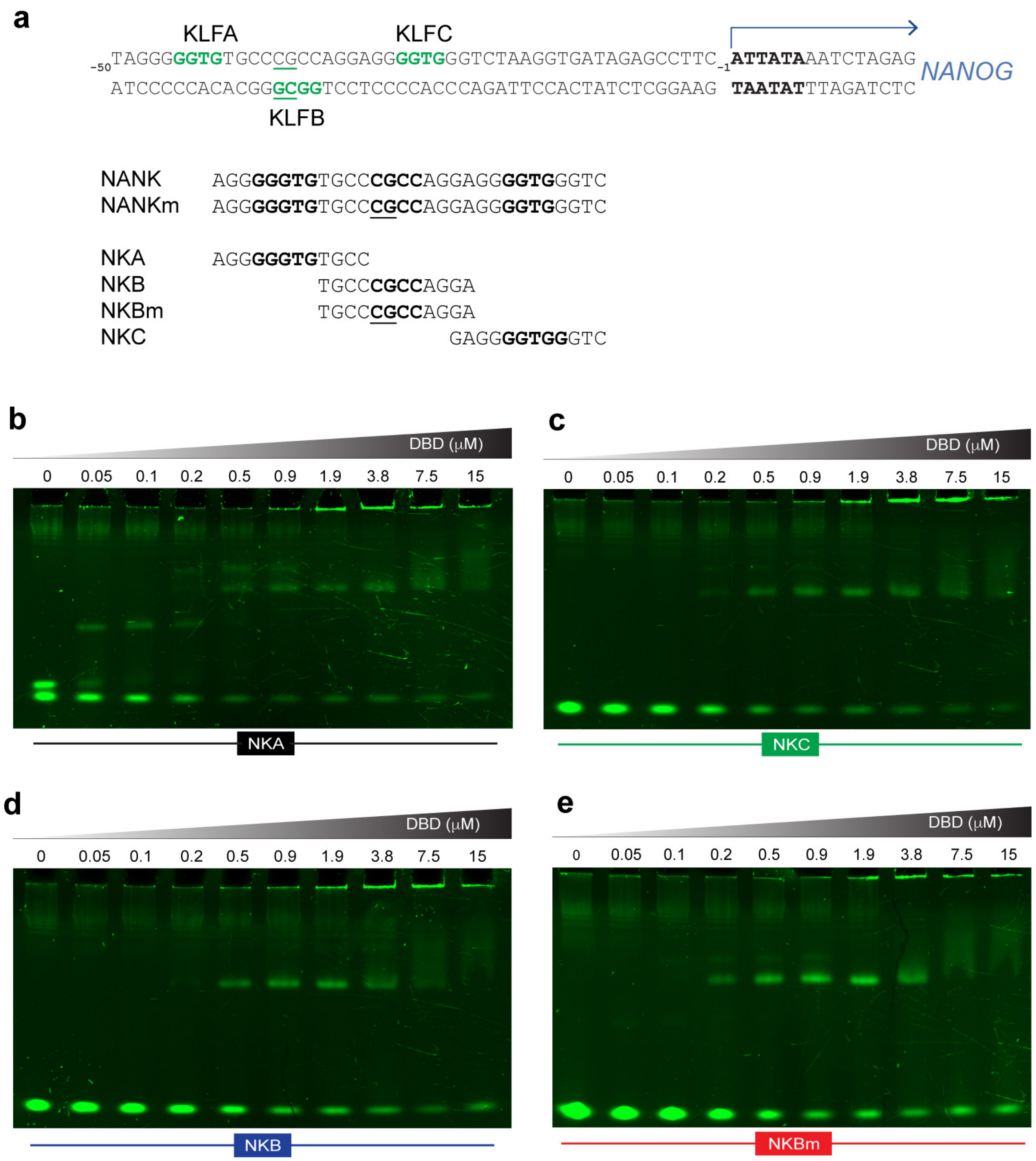

Supplementary Figure 4. **Electromobility shift assays reveal KLF4 DBD binding to single KLF4 cognate sites excerpted from the *NANOG* proximal promoter. a** *NANOG* proximal promoter diagram indicating the three putative KLF4 binding sites and the sequences of DNA oligonucleotide duplexes used to test the ability of each independent site to interact with KLF4 DBD. Electrophoretic mobility shift assays (EMSA) of DBD for **b** NKA, **c** NKC, **d** NKB, and **e** NKBm. EMSAs were performed for 2-3 independent replicates. The EMSA binding reactions consisted of 1x EMSA buffer (0.01 mg/mL BSA, 0.1 mM DTT, 0.05 mM TCEP, 5% (v/v) glycerol, 50 mM NaCl, 20 mM Tris, pH 8), 50-100 nM unlabeled DNA, and serially diluted protein (15 μM at the highest concentration). Gels were stained with SyBr green (Invitrogen). Additional bound bands for NKA in **b** may be due to the presence of 2 bands for DNA alone. Annealed duplex oligos were purchased from Integrated DNA Technologies (IDT).

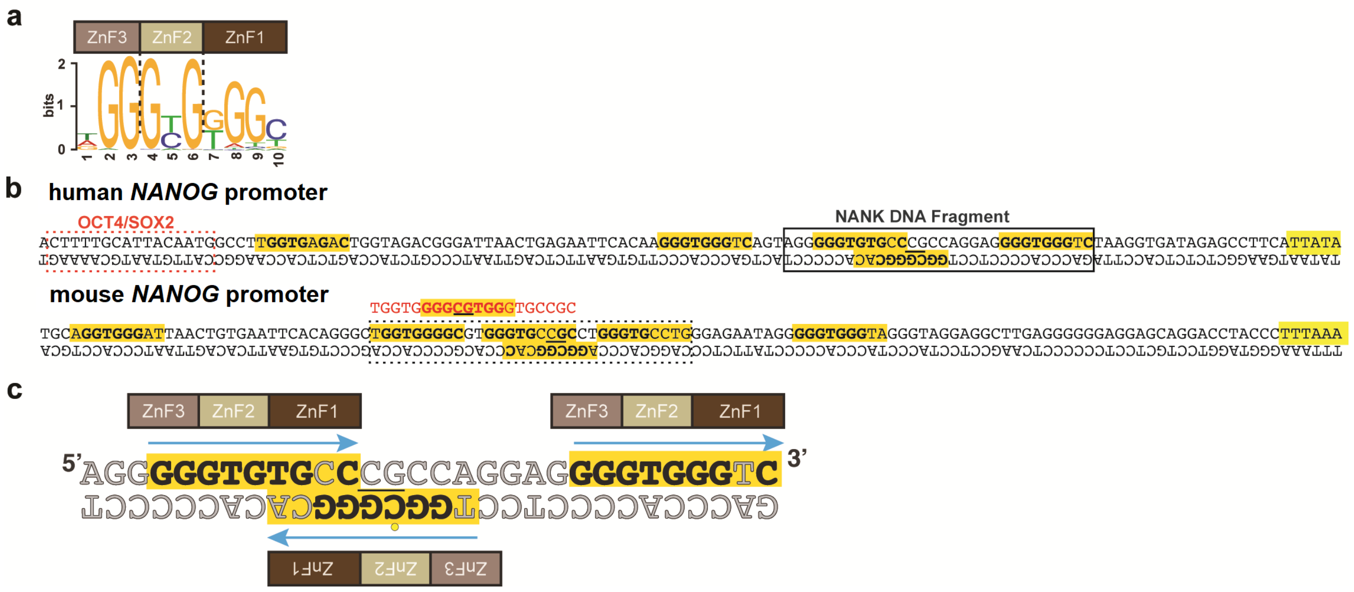

Supplementary Figure 5. **Arrangement of KLF4 sites in human and mouse *NANOG* proximal promoters. a** KLF4 consensus binding site from JASPAR http://jaspar.genereg.net/matrix/MA0039.2/ schematically aligned with the ZnFs that contact each base. **b** Sequence of the human (top) and mouse (bottom) *NANOG* promoter with the G-rich strand of the KLF4 binding sites highlighted in yellow; bases that match the 9 bp consensus are in bold. The OCT4 and SOX2 binding sites (red dashed box) are located upstream of KLF4 binding sites and the TATA box. Overlapping KLF4 sites are present in the human NANK sequence (solid box) and in a region slightly more upstream in mouse (dashed box). The cluster of KLF4 sites include overlap on the same strand, which is depicted by highlighting the 9 bp motif in a second copy of the mouse promoter DNA sequence (in red, above the dashed box). CpG methylation sites are underlined. **c** Schematic diagram for how each KLF4 DBD ZnF is expected to contact the NANK sequence (solid box in **b**) indicates that two ZnF1s would be expected to contact the same bases in the major groove. Blue arrows show the 5’-to-3’ orientation of the G-rich strand of the KLF4 target sequence.

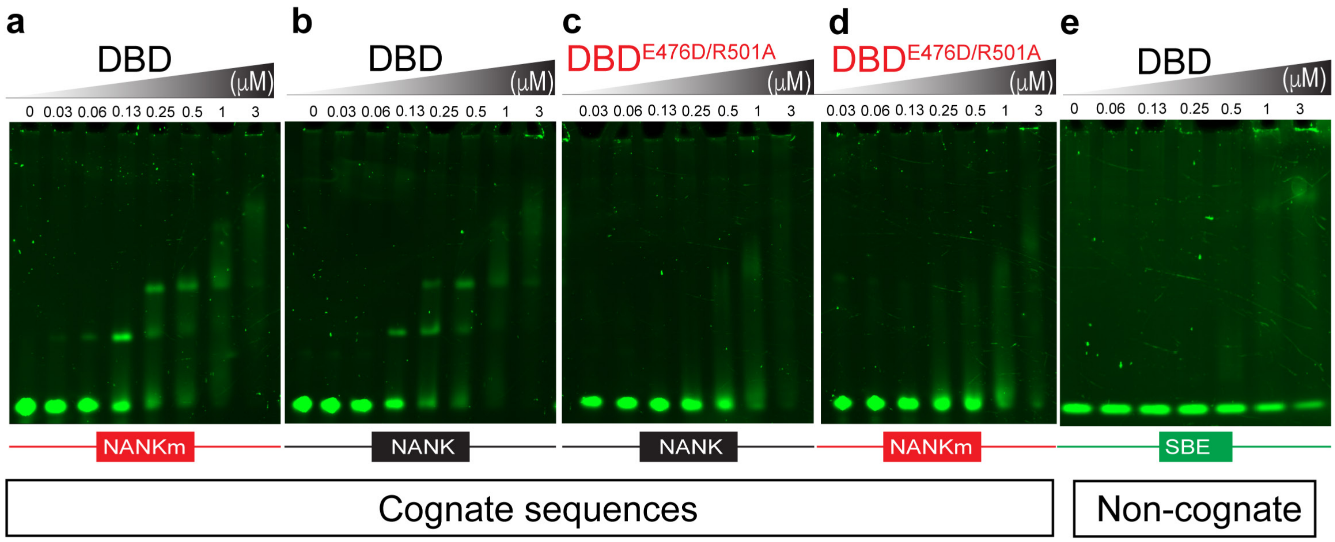

Supplementary Figure 6. **The E476D/R501A double mutant KLF4 DBD binds NANK and NANKm duplexes more weakly than wildtype DBD. a** EMSA of wildtype DBD with methylated NANKm. **b** EMSA of wildtype DBD with NANK. **c** EMSA of double mutant $DBD^{E476D/R501A}$ with NANK. **d** EMSA of double mutant $DBD^{E476D/R501A}$ with methylated NANKm. **e** EMSA of wildtype DBD with non-cognate DNA duplex SBE. EMSAs were replicated 2-3 times.

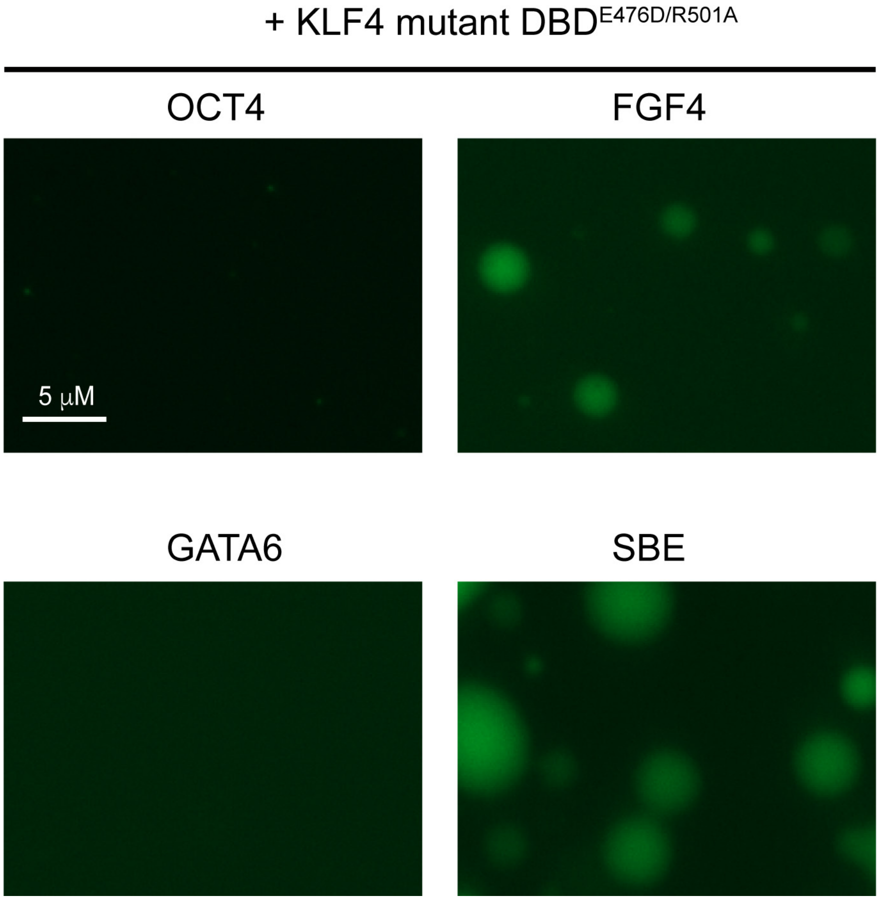

Supplementary Figure 7. **DBD liquid-liquid phase separation with non-cognate DNA duplexes is attenuated by the ZnF 'specificity residue' mutations E476D/R501A.** Fluorescence microscopy images of KLF4 double mutant DBD$^{E476D/R501A}$ with non-cognate DNA duplexes (OCT4, FGF4, GATA6, SBE; oligonucleotide sequences are presented in the bottom section of Supplementary Table 2) at 10 μM DBD$^{E476D/R501A}$ and 3 μM DNA in TS buffer with 100 nM YOYO-1. All images are at the same scale.

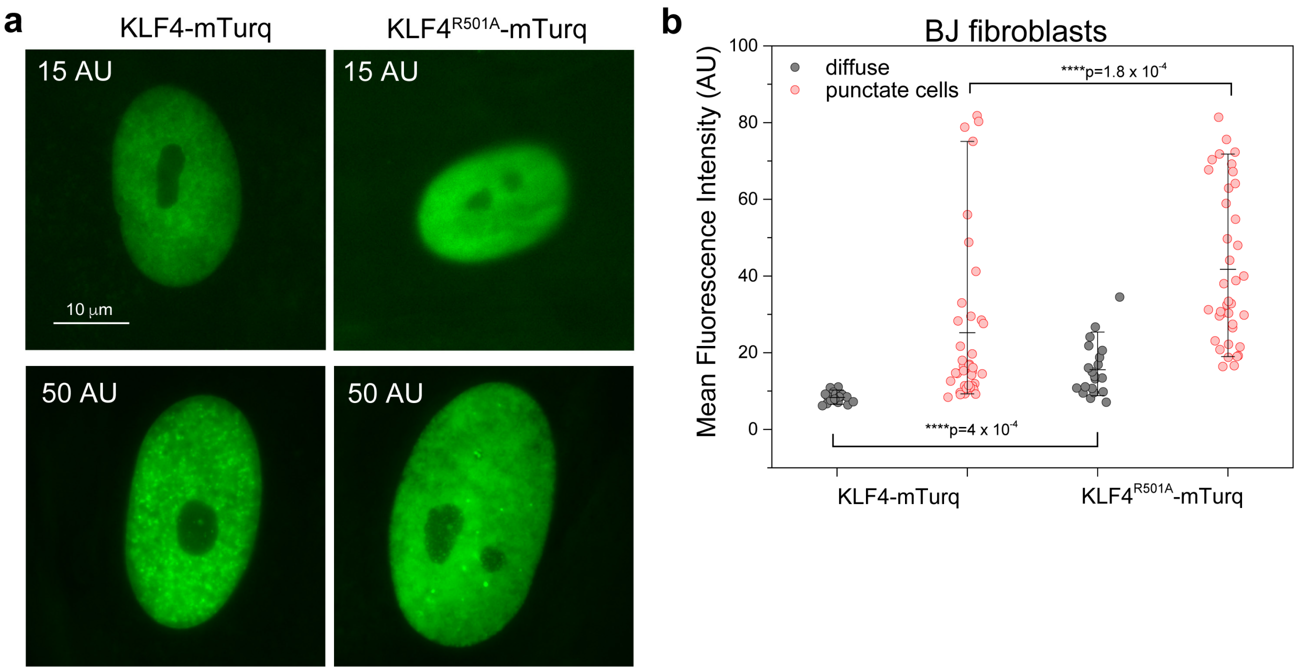

Supplementary Figure 8. **Mutation R501A (specificity position -1 in ZnF2) decreases puncta formation in BJ fibroblasts. a** Fluorescence microscopy of WT KLF4-mTurq and mutant $KLF4^{R501A}$-mTurq expressed in BJ fibroblast cells, compared with same mean fluorescent intensities (15 arbitrary units (AU) in top panels and 50 arbitrary units (AU) in bottom panels; measured by ImageJ). All images taken with EVOS microscopy system at the same scale.

b Plot of the cell distributions of WT and mutant $KLF4^{R501A}$-mTurq. Quantitation from 20 diffuse cells and 38 punctate cells. Mean fluorescent intensities were determined using ImageJ. Punctate cells have at least 5 distinct puncta/droplets (at least 0.5 μM diameter spots by Imaris). The long central horizontal bars mark the median, and the top and bottom bars on the vertical lines denote the 90-10 percentiles. Statistical tests were performed using student's paired t-test; P values are shown). The mutant construct shows the diffuse phenotype at higher expression levels than wild type, and the expression threshold for the punctate phenotype is higher for the mutant than for wild type.

Supplementary Table 1. LIST OF OLIGONUCLEOTIDES		
**OLIGONUCLEOTIDE PRIMERS for PCR and plasmid construction**		
eGFP-F	FOR	5’-AGC TTG CCA CCA TGG GAT CCA GTG GTA CCG AAA ATC TGT ATT TTC AGG GCA TG-3’
eGFP-R	REV	5’-AAC CAC CGG AAC CTC CCT TGT ACA GCT CGT CCA TGC C-3’
pHRT-1F	FOR	5’-GGA GGT TCC GGT GGT TCC G-3’
pHRT-1R	REV	5’-CCC ATG GTG GCA AGC TTG AAT-3’
mTu-1F	FOR	5’-ATC TGT ATT TTC AGG GCA TGG TGA GCA AGG GCG AGG A-3’
mTu-1R	REV	5’-AAC CAC CGG AAC CTC CCT TGT ACA GCT CGT CCA TGC C-3’
pHRT-2R	REV	5’-GCC CTG AAA ATA CAG ATT TTC GGT-3’
KLF4-1F	FOR	5’-GGA TAG GGA TCC CGT CAG CCT CCG GGT GAA AG-3’
KLF4-1R	REV	5’-GCA TTG GGT ACC AAA ATG GCG TTT CAT ATG CAG TG-3’
KLF4-2F	FOR	5’-AAG CTT GCC ACC ATG GGA CGT CAG CCT CCG GGT GAA AG-3’
KLF4-2R	REV	5’-AAT ACA GAT TTT CGG TAC CAC GTT TCG GTT TCG GTT CTT CC-3’
KLF4-3F	FOR	5’-AAG CTT GCC ACC ATG GGA GGT CGT CGT AGC TGG CCT C-3’
KLF4-3R	REV	5’-AAT ACA GAT TTT CGG TAC CAA AAT GGC GTT TCA TAT GCA GTG-3’
KLF4-4F	FOR	5’-CGC TAG TCC GCC GCT TTA ATC-3’
KLF4-4R	REV	5’-CAG TGC CAG ATG ATC GCT AGC GCT AAA TGC ACG ATC ACA-3’
KLF4-5F	FOR	5’-TGT GAT CGT GCA TTT AGC GCT AGC GAT CAT CTG GCA CTG-3’
KLF4-5R	REV	5’-GAT TAA AGC GGC GGA CTA GCG-3’
KLF4-6R	REV	5’-ACG ATA ATG ACG GGT CAG GTC ATC ACT ACG TGC AAA TTT C-3’
KLF4-7F	FOR	5’-GAA ATT TGC ACG TAG TGA TGA CCT GAC CCG TCA TTA TCG T-3’
OCT4-1F	FOR	5'-AAG CTT GCC ACC ATG GGT GCG GGA CAC CTG GCT TCG GA-3'
OCT4-1R	REV	5'-AAT ACA GAT TTT CGG TAC CGT TTG AAT GCA TGG GAG AGC C-3'
OCT4-2F	FOR	5'-CAA CAA GCT GTC TAG AGA ATT CA-3'
OCT4-2R	REV	5'-AAT ACA GAT TTT CGG TAC CGT TTG-3'
SOX2-1F	FOR	5'-CAA GTG GAG CAA GGC AGA CCA TGG GCT ACA ACA TGA TGG A-3'
SOX2-1R	REV	5'-TTA GAC ACC ATG GTA GCC ATG TGT GAG AGG GGC AGT GT-3'
pHRT-3F	FOR	5'-GCT ACC ATG GTG TCT AAA GGC-3'
pHRT-3R	REV	5'-CTG CCT TGC TCC ACT TGA CG-3'
KLF4-8F	FOR	5’-GTA TTT TCA GAG CCA TAT GGG TCG TCG TAG CTG GCC TCG-3’
KLF4-8R	REV	5’-GCC GGA TCT CGA GCT AAA AAT GGC GTT TCA TAT GCA GTG C-3’
pET15-1F	FOR	5’-TAG CTC GAG ATC CGG CTG CTA-3’
pET15-1R	REV	5’-CAT ATG GCT CTG AAA ATA CAG GTT-3’
mTu-2F	FOR	5’-CTT TAA GAA GGA GAT ATA CCA TGG GTA CCG AAA ATC TGT ATT TTC AG-3’
mTu-2R	REV	5’-TGT TAG CAG CCG GAT CTC GAG TGA TCA TTA GTG ATG GT-3’
KLF4-9F	FOR	5’-GAT CGT GCA TTT AGC GCT AGC GAT CAT CTG GC-3’
KLF4-10F	FOR	5’-TTG CAC GTA GTG ATG ATC TGA CCC GTC ATT ATC-3’
KLF4-11F	FOR	5’-GAA GGA GAT ATA CCA TGC GTC AGC CTC CGG GTG AAA G-3’
KLF4-11R	REV	5’-TAC AGA TTT TCG GTA CCA CGT TTC GGT TTC GGT TCT TCC-3’
KLF4-12F	FOR	5’-GAA GGA GAT ATA CCA TGG GTC GTC GTA GCT GGC CTC-3’
KLF4-12R	REV	5’-TAC AGA TTT TCG GTA CCA AAA TGG CGT TTC ATA TGC AGT G-3’
pET15-2F	FOR	5’- GGT ACC GAA AAT CTG TAT TTT CAG-3’
pET15-2R	REV	5’- CAT GGT ATA TCT CCT TCT TAA AGT-3’
hNan-F1	FOR	5’-[AmC6]-GCT GGT TTC AAA CTC CTG ACT TC-3’
hNan-R1	REV	5’-TCC TGG AGT CTC TAG ATT TAT AAT G-3’
hNan-F2	FOR	5’-GCT GGT TTC AAA CTC CTG ACT TC-3’
hNan-R2	REV	5’-[AmC6]-TCC TGG AGT CTC TAG ATT TAT AAT G-3’
**OLIGONUCLEOTIDE DUPLEXES for biochemical assays and biophysics**		
NKA duplex		5’-AGG GGG TGT GCC-3’
NKB duplex		5’-T GCC CGC CAG GA-3’
NKC duplex		5’-GAG GGG TGG GTC-3’
NKBm duplex		5’-T GCC /iMe-dC/GC CAG GA-3’
NANK duplex		5’-AGG GGG TGT GCC CGC CAG GAG GGG TGG GTC-3’
NANKm duplex		5’-AGG GGG TGT GCC /iMe-dC/GC CAG GAG GGG TGG GTC-3’
A488-NANK duplex		(Alexa Fluor 488 labeled) 5’-[AmC6]- AGG GGG TGT GCC CGC CAG GAG GGG TGG GTC -3’
A594-NANK duplex		(Alexa Fluor 594 labeled) 5’-[AmC6]- AGG GGG TGT GCC CGC CAG GAG GGG TGG GTC -3’
GATA6 duplex		5’- AGA ATA AAG TAA AAA GGT TAA TGG CTG AGG GTG GGCC TCA -3’
FGF4 duplex		5’-TTC TTT GTT TGG ATG CTA ATG GGA-3’
OCT4 duplex		5’-GGC CCA TTC AAG-3’
SBE duplex		5’-AAT CAG TCT AGA CAT AC-3’
