# Supplementary material for: Liquid condensation of reprogramming factor KLF4 with DNA provides a mechanism for chromatin organization
Source: Nat Commun. 2021 Sep 22;12:5579. doi: 10.1038/s41467-021-25761-7 (PMC8458463; doi:10.1038/s41467-021-25761-7)
Supplement: Supplementary file 3 — Reporting summary [file 41467_2021_25761_MOESM3_ESM.pdf]

nature research

Corresponding author(s): Dr. Josephine Ferreon

Last updated by author(s): Aug 12, 2021

Reporting Summary

Nature Research wishes to improve the reproducibility of the work that we publish. This form provides structure for consistency and transparency in reporting. For further information on Nature Research policies, see our Editorial Policies and the Editorial Policy Checklist.

Statistics

For all statistical analyses, confirm that the following items are present in the figure legend, table legend, main text, or Methods section.

n/a	Confirmed	
☐	☒	The exact sample size (*n*) for each experimental group/condition, given as a discrete number and unit of measurement
☐	☒	A statement on whether measurements were taken from distinct samples or whether the same sample was measured repeatedly
☐	☒	The statistical test(s) used AND whether they are one- or two-sided<br>*Only common tests should be described solely by name; describe more complex techniques in the Methods section.*
☒	☐	A description of all covariates tested
☒	☐	A description of any assumptions or corrections, such as tests of normality and adjustment for multiple comparisons
☐	☒	A full description of the statistical parameters including central tendency (e.g. means) or other basic estimates (e.g. regression coefficient) AND variation (e.g. standard deviation) or associated estimates of uncertainty (e.g. confidence intervals)
☐	☒	For null hypothesis testing, the test statistic (e.g. *F*, *t*, *r*) with confidence intervals, effect sizes, degrees of freedom and *P* value noted<br>*Give P values as exact values whenever suitable.*
☒	☐	For Bayesian analysis, information on the choice of priors and Markov chain Monte Carlo settings
☒	☐	For hierarchical and complex designs, identification of the appropriate level for tests and full reporting of outcomes
☒	☐	Estimates of effect sizes (e.g. Cohen's *d*, Pearson's *r*), indicating how they were calculated

Our web collection on statistics for biologists contains articles on many of the points above.

Software and code

Policy information about availability of computer code

Data collection	The softwares used structural analyses were CCP4 7.0.078, PHENIX 1.11.1-2575-000, Phaser 2.83, Phenix.refine 1.19.1, COOT 0.8.2, PYMOL 2.2.0. For sequence motif analysis, JASPAR 2020. For microscopic imaging, Zen 2.3, Fiji (ImageJ) 1.52c, LSM780/880 laser-scanning confocal microscope and Imaris 9.2. For smFRET data, we used VistaVision (64) 4.2.220.0. Details of data collection are described in Methods.
Data analysis	For data analysis, we used OriginPro 2020 and VistaVision (64) 4.2.220.0. For microscopic imaging, Fiji (ImageJ) 1.52c and Imaris 9.2. Details of data analysis were described in the manuscript and in Methods.

For manuscripts utilizing custom algorithms or software that are central to the research but not yet described in published literature, software must be made available to editors and reviewers. We strongly encourage code deposition in a community repository (e.g. GitHub). See the Nature Research guidelines for submitting code & software for further information.

Data

Policy information about availability of data

All manuscripts must include a data availability statement. This statement should provide the following information, where applicable:

- Accession codes, unique identifiers, or web links for publicly available datasets
- A list of figures that have associated raw data
- A description of any restrictions on data availability

Source data for plots, raw data for counts and intensity measurements, and uncropped gel images generated in this study are provided in a Source Data file. The structure factors and coordinates for the KLF4 DBD:KLFA structure have been deposited in the Protein Data Bank under the accession number 6vtx.

Field-specific reporting

Please select the one below that is the best fit for your research. If you are not sure, read the appropriate sections before making your selection.

- [x] Life sciences
- [ ] Behavioural & social sciences
- [ ] Ecological, evolutionary & environmental sciences

For a reference copy of the document with all sections, see nature.com/documents/nr-reporting-summary-flat.pdf

Life sciences study design

All studies must disclose on these points even when the disclosure is negative.

Sample size	The sample sizes were ~50-100 cells (each biological replicate) for the cell imaging experiments, consistent with those reported in the literature (e.g., Mann et al, Neuron, (2019) 102,1-18)
Data exclusions	There were no data exclusions.
Replication	All attempts at replication were successful. There were 2-3 independent replicate experiments for in vitro and in cell studies.
Randomization	No randomization was explicitly performed because the experiments (in vitro droplet and cellular imaging) were highly reproducible and independently performed by several researchers.
Blinding	Blinding was not performed because analysis is required, consistent with typical practice in biophysical experiments.

Reporting for specific materials, systems and methods

We require information from authors about some types of materials, experimental systems and methods used in many studies. Here, indicate whether each material, system or method listed is relevant to your study. If you are not sure if a list item applies to your research, read the appropriate section before selecting a response.

Materials & experimental systems

n/a	Involved in the study	
x		Antibodies
	x	Eukaryotic cell lines
x		Palaeontology and archaeology
x		Animals and other organisms
x		Human research participants
x		Clinical data
x		Dual use research of concern

Methods

n/a	Involved in the study	
x		ChIP-seq
x		Flow cytometry
x		MRI-based neuroimaging

Eukaryotic cell lines

Policy information about cell lines

Cell line source(s)	The HEK 293T cell line and BJ fibroblast cells were obtained from ATCC and Lenti-X 293T were obtained from TaKaRa Bio.
Authentication	Authentication from the source (STR profiling)
Mycoplasma contamination	All cells used in this study tested negative for mycoplasm contamination.
Commonly misidentified lines (See ICLAC register)	No commonly misidentified lines used
